# Interaction between circulating galectin-3 and cancer-associated MUC1 enhances tumour cell homotypic aggregation and prevents anoikis

**DOI:** 10.1186/1476-4598-9-154

**Published:** 2010-06-18

**Authors:** Qicheng Zhao, Monica Barclay, John Hilkens, Xiuli Guo, Hannah Barrow, Jonathan M Rhodes, Lu-Gang Yu

**Affiliations:** 1 Gastroenterology Research Unit, School of Clinical Sciences, Centre for Glycobiology, University of Liverpool, Liverpool L69 3GE, UK; 2 Division of Molecular Genetics, the Netherlands Cancer Institute, Amsterdam 1066 CX, Netherlands

## Abstract

**Background:**

Formation of tumour cell aggregation/emboli prolongs the survival of circulating tumour cells in the circulation, enhances their physical trapping in the micro-vasculature and thus increases metastatic spread of the cancer cells to remote sites.

**Results:**

It shows here that the presence of the galactoside-binding galectin-3, whose concentration is markedly increased in the blood circulation of cancer patients, increases cancer cell homotypic aggregation under anchorage-independent conditions by interaction with the oncofetal Thomsen-Friedenreich carbohydrate (Galβ1,3GalNAcα-, TF) antigen on the cancer-associated transmembrane mucin protein MUC1. The galectin-3-MUC1 interaction induces MUC1 cell surface polarization and exposure of the cell surface adhesion molecules including E-cadherin. The enhanced cancer cell homotypic aggregation by galectin-MUC1 interaction increases the survival of the tumour cells under anchorage-independent conditions by allowing them to avoid initiation of anoikis (suspension-induced apoptosis).

**Conclusion:**

These results suggest that the interaction between free circulating galectin-3 and cancer-associated MUC1 promotes embolus formation and survival of disseminating tumour cells in the circulation. This provides new information into our understanding of the molecular mechanisms of cancer cell haematogenous dissemination and suggests that targeting the interaction of circulating galectin-3 with MUC1 in the circulation may represent an effective therapeutic approach for preventing metastasis.

## Introduction

Formation of tumour cell aggregation/emboli in the circulation prolongs the survival of tumour cells and allows their physical trapping in the micro-vasculature and contributes to cancer cell hematogenous dissemination [1,2]. The formation of tumour emboli is heavily regulated by the expression, availability and activity of the cell surface adhesion molecules.

Galectin-3 is a multi-functional galactoside-binding protein that is expressed by many types of human cells. It is found inside and outside of the cells as well as in the circulation. Intracellular galectin-3 is an apoptosis inhibitor [3] and mRNA splicing promoter [4] whilst cell surface-associated extracellular galectin-3 acts as an adhesion molecule in cell-cell interactions [5,6] and promotes cancer progression and metastasis [7,8]. The concentration of free circulating galectin-3 is markedly increased in the sera of patients with breast, colorectal, lung [9], head and neck [10] cancers and melanoma [11]. Patients with metastatic disease are seen to have higher concentrations of circulating galectin-3 than those with localized tumours. Recently, we have shown that the transmembrane mucin protein MUC1 is an endogenous ligand of galectin-3 in human colon cancer cells and that the interaction between MUC1 and galectin-3 occurs via binding of galectin-3 to the oncofetal Thomsen-Friedenreich carbohydrate (Galβ1,3GalNAcα-, T or TF) antigen on MUC1 [12].

MUC1 is a large and heavily glycosylated transmembrane mucin protein that is expressed on the apical surface of normal secretory epithelia [13]. In epithelial cancer cells, MUC1 is over-expressed [14] and aberrantly glycosylated with short oligosaccharides such as GalNAcα- (Tn), sialylated GalNAcα- (sialyl-Tn) and TF antigen [15,16]. On cancer cells, MUC1 also loses its apical polarization and becomes expressed over the entire cell surface [17,18]. TF antigen is covered in normal epithelium by extensive glycosylation, sulphation and/or sialylation but expressed in unsubstituted form by most human cancer cells [19,20]. The increased expression of MUC1 and the increased occurrence of TF antigen are both associated, independently, with high metastatic potential of the cancer cells and poor prognosis of the patients [21,22]. MUC1 is an extremely elongated molecule which protrudes over 10 times further from the cell surface than the typical cell surface adhesion molecules [14] and therefore influences cell adhesion when is present at high density at the cell surface [23]. Thus, over-expression of MUC1 promotes tumour cell release from primary tumour sites by inhibiting E-cadherin-mediated cell-cell and integrin-mediated cancer-extracellular matrix interactions [18,24].

We have previously shown that the interaction between cell surface MUC1 and galectin-3 at concentrations similar to those found in the sera of cancer patients increases cancer cell heterotypic adhesion to endothelium as a result of MUC1 cell surface polarization which leads to exposure of heterotypic cell-cell adhesion molecules that are otherwise concealed by elongated structure of MUC1 [12]. As change of MUC1 cell surface localization in response to galectin-3 binding may also expose the adhesion molecules that are essential to homotypic cancer cell interactions, we hypothesised that an increased interaction between circulating galectin-3 and cancer-associated MUC1 expressed on the surface of circulating tumour cells in cancer patients may promote the formation of cancer cell aggregates/emboli thus prolongs the survival of disseminated tumour cells in the circulation and contributes to cancer cell haematogenous dissemination.

We provide evidence in this study showing that the interaction between cell surface MUC1 and recombinant galectin-3 at pathologically-relevant circulating galectin-3 concentrations increases homotypic aggregation of human colon and breast cancer cells as a result of cell surface clustering of MUC1 and consequent exposure of the homotypic cell adhesion molecules including E-cadherin. The galectin-3-MUC-induced cell aggregation increases the survival of the cancer cells by preventing initiation cellular anoikis (suspension-induced apoptosis).

## Results

### Human colon and breast cancer cell-cell homotypic aggregation is inhibited by MUC1 expression but restored by MUC1-galectin-3 interaction

**Figure 1:**
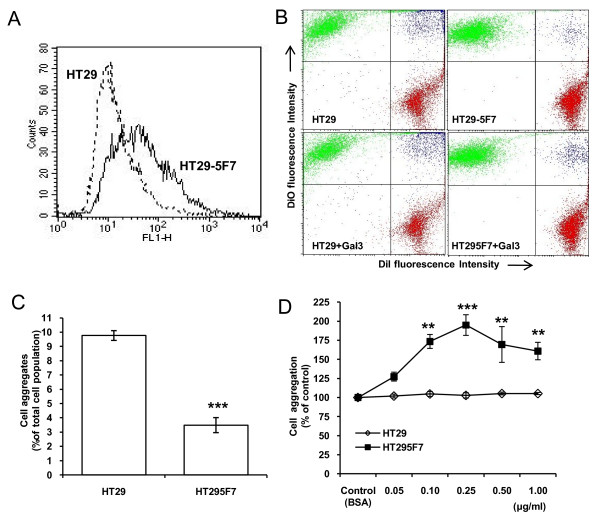
**MUC1 expression prevents and MUC1-galectin-3 interaction promotes homotypic aggregation of human colon cancer cells**. **A**: Cell surface staining with B27.29 anti-MUC1 antibody followed by analysis with flow cytometry shows higher cell surface MUC1 expression in HT29-5F7 than in HT29 cells. **B**: Representative flow cytometry plots from the aggregation assessment of human colon cancer HT29-5F7 and HT29 cells in the presence or absence of 1 μg/ml recombinant galectin-3. The top right (blue) in the bivariate correlation plot are the cell population containing both DiO- and Dil-labelled cells that are defined in this study as cell aggregates. **C**: The more strongly MUC1-expressing HT29-5F7 cells show less spontaneous cell aggregation than the parental HT29 cells. Data are expressed as mean ± SEM of triplicate determinations from three independent experiments. **D**: galectin-3 treatment induces a dose-dependent increase of HT29-5F7 but not HT29 cell aggregation. Data are expressed as mean ± SEM of triplicate determinations from four independent experiments. **p < 0.01, ***p < 0.001.

To test the effect of MUC1 expression on cancer cell homotypic aggregation, we first compared the adhesive properties of human colon cancer HT29 and HT29-5F7 cells. HT29-5F7 is a subpopulation of HT29 cells and was selected for its resistance to 5-fluorouracil and has much higher MUC1 expression than the parental HT29 cells (Fig 1A) [25]. It was found that spontaneous aggregation of HT29 cells was 2.8-fold greater than that of HT29-5F7 cells (Fig 1B and 1C). Pre-treatment of HT29-5F7 cells with recombinant galectin-3 at similar circulating galectin-3 concentrations found in the sera of colorectal cancer patients [9] resulted in a dose-dependent increase of HT29-5F7 but not HT29 cell aggregation (Fig 1D). At 1.0 μg/ml, galectin-3 caused a 69% increased aggregation [169.2 ± 24.7 (mean ± SEM), p < 0.01] of HT29-5F7 but not HT29 cells (91.4 ± 5.9, p = 0.2) (Fig 1B)

**Figure 2:**
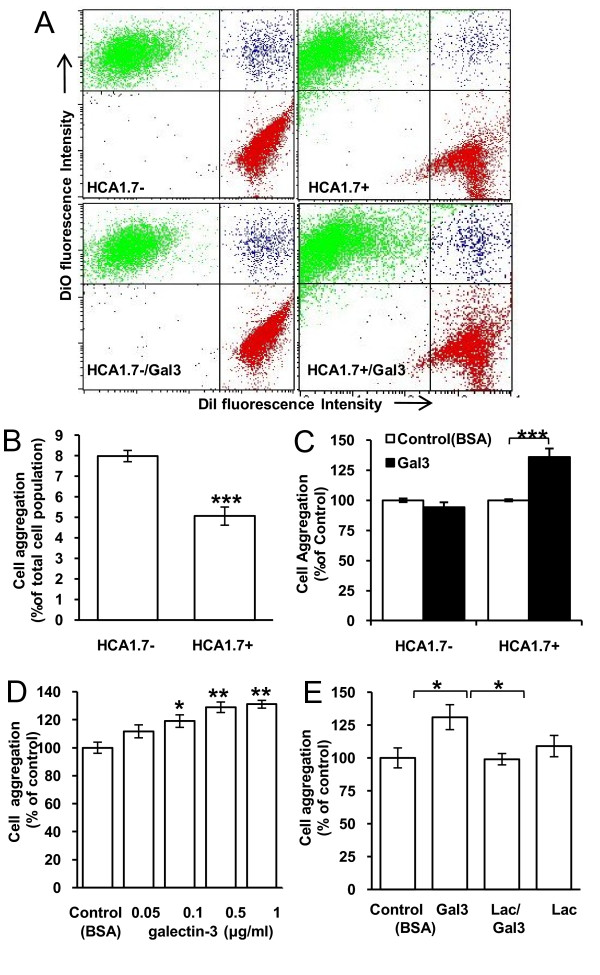
**MUC1 expression prevents and MUC1-galectin-3 interaction promotes homotypic aggregation of MUC1 positively but not negatively transfected human breast epithelial cells**. **A**: Representative flow cytometry plots from the aggregation assessment of MUC1 positive-transfectants (HCA1.7+) and negative-revertants (HCA1.7-) of HBL-100 human breast epithelial cells in the presence or absence or 1 μg/ml recombinant galectin-3. **B**: HCA1.7+ cells show less spontaneous cell-cell aggregation than HCA1.7- cells. Data are expressed as mean ± SEM of triplicate determinations from four independent experiments. **C**: Galectin-3 (1 μg/ml) increases HCA1.7+ but not HCA1.7- cell aggregation. Data are expressed as mean ± SEM of triplicate determinations from four independent experiments. **D**: Galectin-3 induces dose-dependent aggregation of HCA1.7+ cells. Data are expressed as mean ± SEM of triplicate determinations from three independent experiments. **E**: The presence of lactose (10 μM) blocks the increase of HCA1.7+ cell aggregation induced by 1 μg/ml galectin-3. Data are expressed as mean ± SEM of triplicate determinations from two independent experiments. *p < 0.05, **p < 0.01, ***p < 0.001.

**Figure 3:**
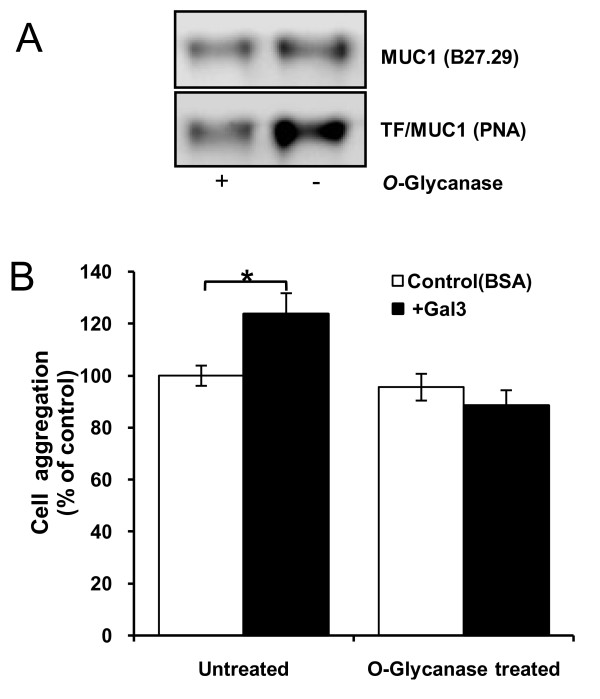
**Effects of TF expression on galectin-3-mediated cell aggregation**. **A**: *O*-Glycanase treatment of HCA1.7+ cells reduces TF-expression on MUC1. **B**: Reduction of TF-expression by *O*-glycanase treatment abolishes the effect of galectin-3 (1 μg/ml) on HCA1.7+ cell aggregation. Data are expressed as mean ± SEM of triplicate determinations from two independent experiments. *p < 0.05.

Spontaneous aggregation of HBL-100 human breast epithelial cells transfected with MUC1-cDNA (HCA1.7+) (5.1 ± 0.4%) was significantly less compared to MUC1-negative controls (HCA1.7-) (8.0 ± 0.3, p < 0.001) (Fig 2A and 2B). Pre-treatment of the cells with galectin-3, at 1.0 μg/ml, caused 36% increased aggregation of HCA1.7+ (136.6 ± 7.0, p < 0.01) but not HCA1.7- cells (94.2 ± 4.2, p = 0.2) (Fig 2C). The effect of galectin-3 on HCA1.7+ cell aggregation was dose-dependent (Fig 2D) and was prevented by the presence of 10 μM lactose (Fig 2E). Pre-treatment of HCA1.7+ cells with *streptococcal O*-glycanase, that specifically removes the unsubstituted TF disaccharide, caused a 58% reduction of TF epitope on MUC1 as assessed by PNA blotting (Fig 3A) and attenuated the galectin-3-induced cell aggregation (Fig 3B). Thus, expression of MUC1 prevents homotypic cell aggregation but the aggregation is restored by the interaction of galectin-3 with TF/MUC1.

### Galectin-3 and B27.29 anti-MUC1 mAb have similar effects on MUC1 cell surface polarization and on epithelial cancer cell-cell aggregation

**Figure 4:**
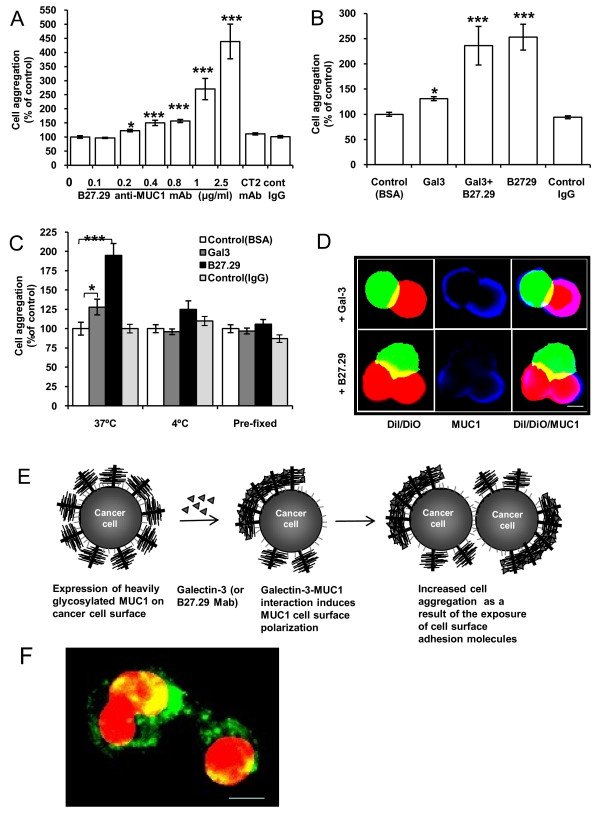
**Galectin-3 and B27.29 anti-MUC1 mAb both induce increase of cell aggregation**. **A**: B27.29 mAb induces dose-dependent increase of HCA1.7+ cell aggregation. HCA1.7+ cell aggregation was determined after pre-incubation of the cells with or without various concentrations of B27.29 Mab, 1 μg/ml control mouse immunoglublin or CT-2 anti-MUC1 antibody. Data are expressed as mean ± SEM of triplicate determinations from three independent experiments. **B**: B27.29 mAb increases HCA1.7+ cell aggregation regardless of the presence or absence of recombinant galectin-3. HCA1.7+ cell aggregation was determined after pre-incubation of the cells with or without 1 μg/ml recombinant galectin-3 in the presence or absence of 1 μg/ml B27.29, BSA or control immunoglublin. Data are expressed as mean ± SEM of triplicate determinations from three independent experiments. **C**: Galectin-3 or B27.29 mAb at 1 μg/ml fails to induce HCA1.7+ cell aggregation at 4°C or to paraformaldehyde-prefixed cells. Data are expressed as mean ± SEM of triplicate determinations from four independent experiments. *p < 0.05,**p < 0.01, ***p < 0.001. **D**: MUC1 localization in cell aggregates. Separate aliquots of HCA1.7+ cells pre-labelled with DiO (green) or DiI (red) were mixed in the presence of 1 μg/ml galectin-3 or B27.29 mAb and incubated for 1 hr at 37°C before fixation and subsequent analysis of MUC1 localization by immunohistochemistry. Representative images of the MUC1 localization in cell aggregates are shown. **F**: localization of the cell aggregates-associated MUC1 (green) after treatment of the cells with 1 μg/ml galectin-3 for 48 hr under suspension (red: cell nucleuses). Bar = 10 μm.

Our previous study demonstrated that B27.29 anti-MUC1 mAb, directed against the PDTRPAP epitope [26] within the VNTR region of MUC1, induces MUC1 cell surface polarization and increases human melanoma cell adhesion to endothelium [27]. We found here that addition of B27.29 mAb to HCA1.7+ cells in suspension also caused a dose-dependent increase in cell aggregation (Fig 4A), regardless of the presence or absence of recombinant galectin-3 (Fig 4B). When the cell aggregates were further analysed, it was found that B27.29 mAb not only induced more cell aggregates but also larger aggregates than recombinant galectin-3 (data not shown).

Fourteen percent (71/500) of the HCA1.7+ cells showed spontaneous clustering of MUC1 on the cell surface, when cultured in suspension for 1 hour at 37°C. After pre-incubation of the cells with 1 μg/ml recombinant galectin-3 or 27.29 mAb for 1 hr at 37°C, 57% (112/500, P < 0.01) and 93% (133/500, P < 0.01) more cells, respectively, than the control BSA-treated cells demonstrated MUC1 cell surface clustering. Introduction of galectin-3 or mAb B27.29 to the cells at 4°C or to paraformaldehyde pre-fixed cells had no effect on MUC1 localization and no significant effect on cell aggregation compared with the control BSA-treated cells (Fig 4C).These results indicate a direct link between discontinuous cell surface localization of MUC1 and the increased cell aggregation in response to galectin-3 and B27.29 mAb. In keeping with this, MUC1 was observed to be absent at the cell-cell contact point within the cell aggregates in the cells treated either with galectin-3 or mAb B27.29 (Fig 4D). Thus, polarization of cell surface MUC1 in response to galectin-3 or anti-MUC1 antibody binding exposes the smaller cell surface adhesion molecules that increases homotypic cell aggregation (Fig 4E). The higher cell aggregation induced by B27.29 antibody than by galectin 3 is in keeping with the notion that a lectin-glycan interaction is weaker than an antibody-antigen interaction. We have observed that after 48 hr treatment with galectin-3 under suspension the cell aggregates-associated MUC1 molecules were still clustered on the cell surface with some degree of internalization and degradation (Fig 4F).

### Involvement of cell surface E-cadherin in galectin-3-mediated cell aggregation

**Figure 5:**
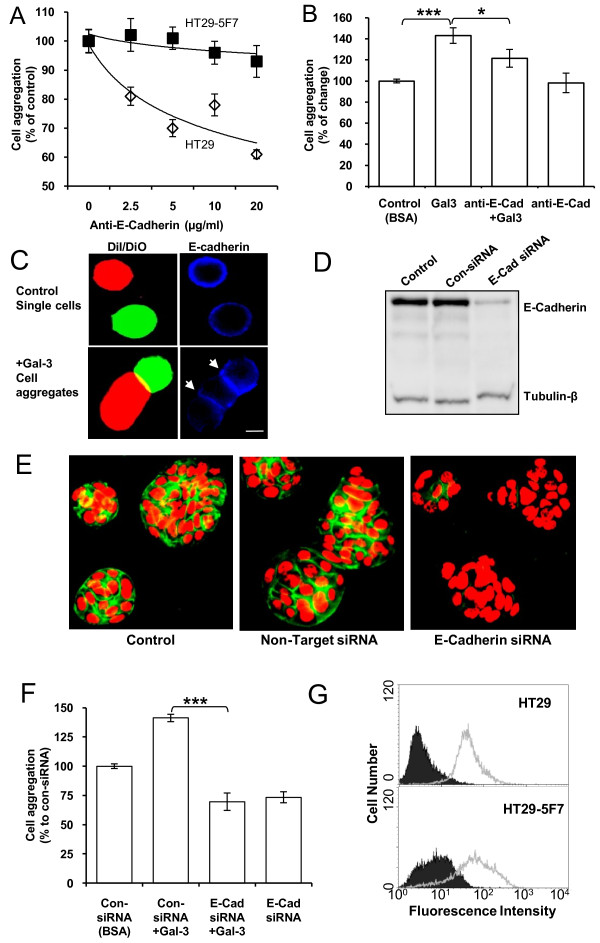
**Involvement of cell surface E-cadherin in galectin-3-induced cell aggregation**. **A**: The presence of anti-E-cadherin antibody reduces spontaneous aggregation of HT29 but has little effect on aggregation of HT29-5F7 cells. Data are expressed as mean ± SEM of triplicate determinations from three independent experiments. **B**: Anti-E-cadherin antibody at 20 μg/ml prevents HT29-5F7 cell aggregation-induced by (1 μg/ml) galectin-3. Data are expressed as mean ± SEM of triplicate determinations from three independent experiments. **C: **E-cadherin localization in single cells and cell aggregates. HT29-5F7 cells released by NECDS and labelled separately with DiO and DiI were mixed in the presence or absence of galectin-3 for 1 hr at 37°C. The cells were fixed, probed with mAb B27.29 anti-MUC1 or anti-E-cadherin and fluorescent-labelled secondary antibody and analysed by fluorescent microscopy. E-cadherin shows localization/accumulation at the cell-cell contact points (arrowed) in the cell aggregates. **D**: Western blot showing that transfection of HT29-5F7 cells with siRNA for E-cadherin suppresses E-cadherin expression. **E: **E-cadherin immunohistochemistry shows marked reduction of the cell surface E-cadherin (green) in HT29-5F7 cells after treatment with E-cadherin siRNA for 72 hr (red: cell nucleus). **F**: SiRNA-mediated knock-down of E-cadherin expression prevents galectin-3-mediated HT29-5F7 cell aggregation. Data are expressed as mean ± SEM of triplicate determinations from three independent experiments. **G: **flow cytometry analysis of HT29 and HT29-5F7 cells stained with the MAB1838 anti-E-cadherin antibody show similar E-cadherin cell surface expressions. Open histogram: E-cadherin; shaded histogram: immunoglobulin isotype control.

E-cadherin is known to play a very important role in epithelial cell-cell interactions [18,24]. It was found here that the presence of 2.5 to 20 μg/ml of MAB1838 anti-E-cadherin mAb caused a dose-dependent reduction of the spontaneous aggregation of HT29 but not HT29-5F7 cells (Fig 5A). However, the presence of 20 μg/ml of this antibody inhibited HT29-5F7 cell aggregation induced by galectin-3 (Fig 5B). This indicates the involvement of E-cadherin in spontaneous aggregation of HT29 cells and in galectin-3-induced HT29-5F7 cell aggregation. This is further supported by the discoveries that E-cadherin was localized at the cell-cell contact points of galectin-3-mediated cell aggregates (Fig 5C) and suppression of E-cadherin expression in HT29-5F7 cells by siRNA (Fig 5D and 5E) resulted in significant reduction of spontaneous cell aggregation and prevented the increase of cell aggregation in response to galectin-3 (Fig 5F).

We found that HT29 and HT29-5F7 cells have similar levels of cell surface E-cadherin and similar anti-E-cadherin antibody accessibility (Fig 5G). Thus, the inability of the anti-E-cadherin antibody to prevent HT29-5F7 cell aggregation is most likely due to a functional "concealment" of the cell surface E-cadherin in HT29-5F7 cells, e.g by the presence of adjacent MUC1. The blockade by the anti-E-cadherin antibody of galectin-3-mediated HT29-5F7 cell aggregation (Fig 5B) is in support of this. The fact that the blockade of anti-E-cadherin antibody on galectin-3-mediated aggregation is only partial implies the likely involvement of other cell surface adhesion molecules, which, like E-cadherin, may be exposed following MUC1 polarization in response to galectin-3 binding.

### Galectin-3-induced cell aggregation allows cells to avoid initiation of anoikis

**Figure 6:**
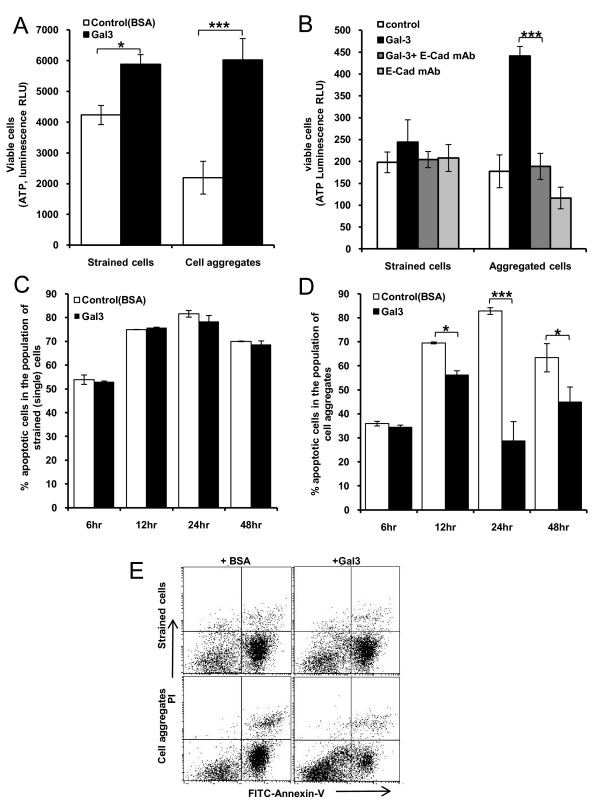
**Galectin-3-induced cell aggregation enhances cell survival by avoiding initiation of anoikis**. **A**: Galectin-3-induced cell aggregation is associated with increased survival of the cells under anchorage-independent conditions. HT29-5F7 cells treated with or without 1 μg/ml galectin-3 or BSA for 1 hr followed by culture of the cells in suspension for 3 days at 37°C. After separation of the cells by cell strainers, the viability of the strained (single) cells and cell aggregates was assessed. The data are presented as mean ± SEM of triplicate determinations from two independent experiments. **B: **The presence of 20 μg/ml anti-E-cadherin antibody inhibited galectin-3-mediated increase of survival of HT29-5F7 cell aggregates. The data are presented as mean ± SEM of triplicate determinations from two independent experiments. **C and D: **Galectin-3 treatment has no effect on anoikis of the single cells (C) but significant reduction of anoikis of the cell aggregates (D). Anoikis was assessed by Annexin-V cell surface binding after treatment of the cells with galectin-3 and subsequent separation of the single cells and cell aggregates. The data are presented as mean ± SEM of triplicate determinations from two independent experiments. **E**. Representative flow cytometry plots from the anoikis assessments of HT29-5F7 cells in the presence of 1 μg/ml galectin-3 or BSA for 48 hr at 37°C. Annexin-V positive and PI negative (early apoptotic, at the bottom right in the bivariate correlation plot) and Annexin-V positive and PI positive (late apoptotic, at the top right in the correlation plot) cells are considered as apoptotic cells.

Nearly 4-fold more cells in the aggregated cell population obtained from the galectin-3-treated cells were viable than those obtained from the BSA-treated controls after 48 hr culture of the cells under suspension conditions (Fig 6A). This indicates that the increase of cell aggregation in response to galectin-3 greatly enhances the survival of the cells under anchorage-independent conditions. A modest 36% increase of the cell viability was also observed in the cell population that passed through the 40 μm Cell Strainers (referred to as "Strained" cells in Fig 6) from the galectin-3-treated cells compared with the BSA-treated controls. As HT29-5F7 cells are 15-20 μm in diameter, it is possible that this small increase is contributed by the inclusion of small cell aggregates, e.g. those formed by 2 or 3 cells, in this cell population. Many small cell aggregates were indeed observed in this population by microscopy (not shown).

We found that the presence of MAB1838 anti-E-cadherin antibody, which prevented galectin-3-induced cell aggregation (Fig 5B), completely inhibited galectin-3-mediated increase of the survival of the cell aggregates (Fig 6B). This suggests that cell surface E-cadherin is critically involved in galectin-3-mediated cell aggregation and survival under anchorage-independent condition.

Assessments of anoikis showed no significant difference between the two strained (single) cell populations obtained from galectin-3-treated and BSA-treated controls within 48 hr culture of the cells under suspension conditions (Fig 6C and 6E). However, significantly fewer cells in the population of cell aggregates obtained from the galectin-3-treated cells were undergoing anoikis than from the BSA-treated controls (Fig 6D and 6E). A 20% (70.0 ± 0.4% *vs *56.1 ± 1.8%, p = 0.02), 65% (82.8 ± 1.4% *vs *28.7 ± 8.0%, p = 0.0001) and 29% 63.4 ± 5.9% *vs *44.9 ± 6.2%, p = 0.02) reduction of anoikis were seen in the cell aggregates obtained from the galectin-3 treated than from the BSA-treated control cells after 12, 24 and 48 hr, respectively (Fig 6D). This indicates that the galectin-3-induced cell aggregation is associated with resistance of those cells to anoikis initiation.

## Discussion

This study shows that the presence of galectin-3 at pathologically-relevant circulating galectin-3 concentrations increases cancer cell homotypic aggregation by interaction with cancer-associated MUC1 that expresses the TF disaccharide. The interaction between galectin-3 and MUC1 causes MUC1 cell surface clustering and exposes smaller cell surface adhesion molecules including E-cadherin. The galectin-3-MUC1-induced cell aggregation enhances the survival of the cells under anchorage-independent conditions by preventing initiation of cellular anoikis. These discoveries have important implications for our understanding of the molecular mechanisms that underlie cancer metastasis.

Survival of tumour cells in the blood/lymphatic circulation is crucial in cancer cell metastatic spread [1,2]. Prolonging the survival of disseminating tumour cells in the circulation directly increases metastasis [28]. Cells in aggregated form have been shown to have a much higher survival rate in the circulation than single cells [29,30]. Thus, an increase of tumour cell aggregation as a result of the increased interaction between circulating galectin-3 and cancer-associated MUC1 in cancer patients provides a survival advantage to the disseminating tumour cells in the circulation. Our results have shown that the enhanced survival of the tumour cells under anchorage-independent conditions by the galectin-3-MUC1 interaction is associated with an enhanced ability of the cell aggregates to escape initiation of anoikis. Anoikis is a specific type of apoptotic process induced by loss of cell adhesion or inadequate cell-matrix interactions [31] and has been proposed to be the dominant mechanism in removing disseminating tumour cells from the circulation [32]. Resistance to anoikis is considered to be a hallmark of metastatic cancer cells [33]. As survival of the tumour cells in the circulation and being eventually able to produce metastasis at remote site occur only in less than 1 in 10,000 of the invaded cells [29,34], the enhanced survival of the disseminating tumour cells by the galectin-3-MUC1 interaction may have profound consequences on the metastatic potential of the cancer cells. The discovery by Topal *et al *[35] that intraportal injection of aggregated DHD/K12/TRb colon cancer cells into syngeneic BD IX rats produces over 4-times more liver metastases than injection with the same numbers of single cells is in consistence with this conclusion.

Furthermore, mechanical as well as "seed-soil" compatibility factors are both believed to contribute to the ability of specific types of cancer cells to spread to various target organs [1,36]. It is thought that the initial docking of specific cancer cells to the target organ is predominantly controlled by mechanical factors and that this is then followed by regulation of specific cell adhesion molecules on the cell surface. Thus, an increase of tumour cell homotypic aggregation as a result of the increased interaction between circulating galectin-3 and cancer-associated MUC1 in the bloodstream of cancer patients is likely to enhance physical trapping of the circulating tumour cells in the microvasculature at target organ and this also enhances metastasis.

It has been shown previously that cell surface-associated galectin-3 acts as a cell adhesion molecule and increases cancer cell homotypic aggregation by interaction with TF antigen expressed by unknown cell surface molecules on adjacent cells under anchorage independent conditions [6,37]. As cell surface-associated galectin-3 is relatively small in size, it is likely that such interaction may occur only after MUC1 cell surface polarization and exposure of the cell surface-associated galectin-3. It should be noted that clustering of the galectin cell surface receptors in response to galectin binding is probably not rare and has been shown to occur in some other cells types such as T cells [38,39]. Although the mechanism of galectin-ligand clustering remains unclear, clustering of galectin-3 ligands has been shown to enhance its binding affinity by as much as 10,000-fold [40].

It should be noted that HT29-5F7 cells also express mRNAs for secreted MUC5AC and MUC5B mucins (with MUC5B the most highly expressed) and transmembrane MUC1 and MUC3 mucins (with MUC1 the most highly expressed) [25]. Although it is unknown whether the transmembrane MUC3 also carries the TF structure and a similar mechanism of interaction between galectin-3 and MUC3 exists, the contribution of such an interaction to galectin-3-mediated cell aggregation in those cells is likely to be much less than that of the galectin-3-MUC1 interaction as the level of MUC1 expression in these cells is much greater than for MUC3.

Thus, the interaction between circulating galectin-3 and TF-expressing MUC1 on the surface of disseminating cancer cells promotes cell aggregation and embolus formation and enhances survival of disseminating tumour cells in the circulation. This, together with our earlier reports showing an enhanced cancer cell-endothelial adhesion also resulting from the galectin-3-MUC1 interaction [12,27], indicates that the increased circulation of galectin-3 in the bloodstream of cancer patients promotes several important steps of the metastatic cascade. The reduction of metastasis-associated survival of nude mice intravenously injected with galectin-3-pretreated than untreated MUC1-expressing human melanoma cells observed in our earlier study [27] likely represents the consequence of galectin-3-MUC1 interaction on cancer cell heterotypic adhesion to endothelium as well as on cancer cell homotypic aggregation. This study provides new information into our understanding of the molecular mechanisms of cancer cell haematogenous dissemination and further highlights the functional importance of change of the cell surface glycosylation in cancer progression. It also implies that targeting the interaction of circulating galectin-3 with MUC1 in the circulation may represent an effective therapeutic approach for preventing metastasis.

## Materials and methods

### Materials

Recombinant full-length human galectin-3 and monoclonal antibody (mAb) against E-cadherin (MAB1838) were from R&D Systems (Abingdon, UK). B27.29 anti-MUC1 mAb was kindly provided by Dr. Mark Reddish (Biomira Inc, Canada). CT-2 anti-MUC1 cytoplasmic tail antibody was from NeoMarkers (Fremont CA). Biotin-conjugated peanut agglutinin (PNA) was purchased from Vector Laboratories Ltd (Peterborough, UK). *Streptococcus pneumoniae *Endo-*N*-acetyl-galactosaminidase (EC 3.2.1.97), *O*-glycanase, was obtained from Prozyme Inc (Oxford, UK). The Non-Enzymatic Cell Dissociation Solution (NECDS) was from Sigma. The Vybrant DiO and DiI Cell-labelling Solutions were from Molecular Probes (Cambridge, UK).

### Cell lines

Human colon cancer HT29 and HT29-5F7 cells were cultured as previously described [12]. Human colon cancer HT29-5F7 cells were kindly provided by Dr. Thecla Lesuffleur (INSERM U560, Lille, France). MUC1 transfection of HBL-100 human breast epithelial cells with full length cDNA encoding MUC1 resulted in the MUC1 positive transfectants HCA1.7+ and the subsequent bulk selection of the MUC1 negative revertants HCA1.7- were as described previously [18].

### Cell aggregation

Subconfluent cells were released from the culture plates with NECDS (which releases the cells while keeping the cell membrane proteins intact) and were dispersed in serum-free DMEM containing 0.5 mg/ml bovine serum albumin (BSA). Two 0.5 ml (1×10^6 ^cells) aliquots of the cell suspension were incubated, separately, with 5 μl/ml DIO and DiI Cell Labelling Solution for 30 min at 37°C. The DIO- and DiI-labelled cells were then mixed in the presence or absence of recombinant galectin-3, antibodies or lactose in DMEM in a rotating incubator (Julabo lab GMBH, Seelbach, Gemerney) at 100 rpm/min at 37°C for 1 hr followed by analysis with flow cytometry (Becton-Dickinson FACSVantage SE). Cells labelled only with DiI or DiO were used to identify the position of DiO- (upper-left panel) and DiI-(bottom-right panel) labelled cell populations in the bivariate correlation plot and cell populations containing both DiO and DiI fluorescence (top-right panel) in the correlation plot were defined as cell aggregates (Fig 1A).

This method measures cell aggregates containing both DiO- and DiI-labelled cells and does not include cell aggregates formed only by DIO- or DiI-labelled cells. Although this may underestimate the total cell aggregates, it provides a more accurate assessment of the effect of exogenous galectin-3 by reducing the variations hence the experimental errors due to the differences of initial existing numbers of cell aggregates in each individual assessment.

### Reduction of TF expression by O-glycanase treatment

HCA1.7+ cells (10^5 ^cells) were incubated with 0.02U/ml O-glycanase for 2 hr at 37°C. The cells were either lysed directly with SDS-sample buffer for blotting with PNA or labelled with DiO and DiI and incubated with recombinant galectin-3 for 1 hr at 37°C before the cell aggregation assessments.

### Electrophoresis and lectin/immunoblotting

SDS-PAGE (4% running gel and 3.5% stack gel for MUC1 blotting and 10% running gel and 4% stack gel for E-cadherin blotting) electrophoresis and immune/lectin blotting were performed as previously described [12].

### MUC1 cell surface localization

Cells released with NECDS were labelled, separately, with DiO and DiI as described above. Two equal cell populations (5×10^5^) of the labelled cells were mixed in the presence or absence of 1 μg/ml galectin-3, B27.29 antibody or BSA for 1 hr at 37°C. The cell suspensions were applied to poly-lysine coated slides, fixed with 2% paraformaldehyde for 15 min, blocked with 5% normal goat serum for 0.5 hr, probed with B27.29 anti-MUC1 antibody (1:2,000 dilution in 1% goat serum/PBS) and followed by fluorescent-labelled secondary antibody (1:400 dilution in 1% goat serum/PBS). The slides were blinded and the number of cells lacking a continuous rim of MUC1 or the number of small or large aggregates in 500 adjacent cells in randomly selected low power fields was counted using an Olympus B51 fluorescent microscope with 20× objective.

In some experiments, HT29 or HT29-5F7 cells released by NECDS were fixed with 2% paraformaldehyde for 15 min, blocked with 5% normal goat serum for 0.5 hr, probed with B27.29 anti-MUC1 antibody (1:2,000 dilution in 1% goat serum/PBS) and followed by analysis of the MUC1 cell surface expression with flow cytometry.

In some other experiments, HT29-5F7 cells released by NECDS were treated with galectin-3 for 1 hr at 37°C before introduced and cultured in poly-HAMA-coated plates for 48 hr. The cells were then fixed with 2% paraformaldehyde for 15 min, blocked with 5% normal goat serum for 0.5 hr, probed with B27.29 anti-MUC1 antibody (1:2,000 dilution in 1% goat serum/PBS) and the MUC1 cell surface localization was observed under fluorescent microscope.

### E-cadherin cell surface expression

Cells released with NECDS were fixed 2% paraformaldehyde for 15 min, blocked with 5% normal goat serum for 0.5 hr, incubated with anti-E-cadherin antibody (1:1000 dilution in 1% goat serum/PBS) or isotype control immunoglobulin and followed with fluorescent-conjugated secondary antibodies (1:400 dilution in 1% goat serum/PBS). The cell surface expression of E-cadherin was analysed by flow cytometry.

### siRNA E-cadherin transfection

HT29-5F7 cells were treated with commercially available siRNA constructs (100 nM) against E-cadherin or scrambled control non-targeting siRNA (siCONTROL non-targeting siRNA, Dhamacon) for 48 hr at 37°C. The cells were either lysed and the expressions of E-cadherin was analysed by immunoblotting or the cells were labelled with DiO and DiI followed by the assessment of cell aggregation in the presence or absence of 1 μg/ml galectin-3 as described above.

In some experiments, the HT29 cells were seeded in 24-well plates with glass coverslips inserted for 18 hr at 37°C before treatment of the cells without or with E-cadherin siRNA or non-targeting control siRNA for 48 to 72 hr. The cells were then fixed with 2% paraformaldehyde for 15 min, blocked with 5% normal goat serum for 0.5 hr, incubated with anti-E-cadherin antibody (1:1000 dilution in 1% goat serum/PBS) for 1 hr. After application of fluorescent-conjugated secondary antibodies (1:400 dilution in 1% goat serum/PBS), the cell surface expression of E-cadherin was observed under fluorescent microscope.

### Cell viability and anoikis in strained and aggregated cells under anchorage-independent condition

The cells were cultured in 96 or 24 well plates that were coated twice with 200 μl/well (96-well plates) or 5 ml/well (6-well plates) of 10 mg/ml poly-2-hydroxyethyl methacrylate (poly-HEMA, Sigma) in 95% ethanol over night at room temperature. After washing, the cells were treated with 1 μg/ml recombinant galectin-3 or BSA under suspension culture in serum-free medium for 6 to 48 hr at 37°C. The cells in suspension were collected and passed three times through a 40-μm cell strainer (BD Biosciences). The viability of the cells that did (strained) and did not (aggregates) pass through the strainers was both measured by the CellTiter-Glo^® ^Luminescent Cell Viability Assay (Promega).

For the measurement of cellular anoikis, after separation of the cells by cell strainers, the strained cells and cell aggregates were resuspended in 0.05% trypsin in PBS and the apoptotic cells were measured by FITC-Annexin-V cell surface binding with a FITC-Annexin-V Apoptosis Detection Kit (Cambridge Biosciences, Cambridge, UK) by flow cytometry as the manufacture's instructions.

### Statistical analysis

The statistical analyses were performed using the unpaired *t *test for single comparison, one-way analysis of variance (ANOVA) followed by Newman and Keuls test for multiple comparisons or Chi-Square test (StatsDirect for Windows, StatsDirect Ltd; Sale, UK) where appropriate. Differences were considered significant when p < 0.05.

## Abbreviations

NECDS: Non-Enzymatic Cell Dissociation Solution; TF antigen: Thomsen-Friedenreich (Galβ1,3GalNAcα-) antigen.

## Competing interests

The authors declare that they have no competing interests.

## Authors' contributions

QZ contributed to acquisition of data and experiment design. MB contributed to data analysis by flow cytometry. JH contributed to the generation of the HCA1.7+/- cells and interpretation of data. XG and HB participated in some experiments. JMR contributed to data analysis and assisted in drafting the manuscript. LGY contributed to conception, design and coordination of the study and drafting the manuscript. All authors read and approved the final manuscript.
